# Aquaporin-4 deletion ameliorates hypoglycemia-induced BBB permeability by inhibiting inflammatory responses

**DOI:** 10.1186/s12974-018-1203-8

**Published:** 2018-05-24

**Authors:** Fei Zhao, Jiangshan Deng, Xiaofeng Xu, Fengya Cao, Kaili Lu, Dawei Li, Xiaojuan Cheng, Xiuzhe Wang, Yuwu Zhao

**Affiliations:** 1 Department of Neurology, Shanghai Jiao Tong University Affiliated Sixth People’s Hospital, No. 600, Yishan Road, Xuhui District, Shanghai, China; 2 School of Pharmacy, Shanghai Jiao Tong University, No. 800, Dongchuan Road, Minhang District, Shanghai, China

**Keywords:** Hypoglycemia, AQP4, BBB, Tight junctions, PPAR-γ, Inflammation

## Abstract

**Background:**

Severe hypoglycemia induces brain edema by upregulating aquaporin-4 (AQP4) expression and by degrading tight junctions. Acute severe hypoglycemia induces a proinflammatory environment that may contribute to a disruption in the epithelial barrier by decreasing tight junction protein expression. Interestingly, the altered AQP4 expression has been considered to play a critical role in neuroinflammation during acute brain injury. It has been shown that AQP4 deletion reduces brain inflammation in AQP4-null mice after intracerebral LPS injection. However, the effect of AQP4 deletion regarding protection against hypoglycemia-induced blood-brain barrier (BBB) breakdown is unknown.

**Methods:**

An acute severe hypoglycemic stress model was established via injection of 4 unit/kg body weight of insulin. Evans blue (EB) staining and water measurement were used to assess BBB permeability. Western blot, reverse transcription polymerase chain reaction, and immunofluorescence were used to detect the expression of related proteins. The production of cytokines was assessed via enzyme-linked immunosorbent assay.

**Results:**

Hypoglycemia-induced brain edema and BBB leakage were reduced in AQP4^−/−^ mice. AQP4 deletion upregulated PPAR-γ and inhibited proinflammatory responses. Moreover, knockdown of aquaporin-4 by small interfering RNA in astrocytes co-cultured with endothelial cells effectively reduced transendothelial permeability and degradation of tight junctions. Treatment with PPAR-γ inhibitors showed that upregulation of PPAR-γ was responsible for the protective effect of AQP4 deletion under hypoglycemic conditions.

**Conclusions:**

Our data suggest that AQP4 deletion protects BBB integrity by reducing inflammatory responses due to the upregulation of PPAR-γ expression and attenuation of proinflammatory cytokine release. Reduction in AQP4 may be protective in acute severe hypoglycemia.

**Electronic supplementary material:**

The online version of this article (10.1186/s12974-018-1203-8) contains supplementary material, which is available to authorized users.

## Background

Hypoglycemia is a threatening complication that frequently occurs in diabetic patients subjected to intensive glycemic control. The brain is the most sensitive organ to hypoglycemia because it highly depends on a continuous supply of glucose from the blood [1]. In addition, concerns have been raised regarding functional brain failures, such as cognitive impairments, seizure, coma, or even death [2–4]. Meanwhile, hypoglycemic damage to the blood-brain barrier (BBB) will have worse results, such as cerebral edema, inflammation, and neuronal injury [5–7]. The importance and increased prevalence of hypoglycemia have been noticed, but the precise molecular mechanisms underlying hypoglycemic BBB breakdown remain to be elucidated.

The integrity of the BBB is maintained by three main elements: brain microvascular endothelial cells (BMECs), astrocytic endfeet, and pericytes. BMECs play a central role in maintaining BBB integrity, which is achieved primarily through the presence of tight junction proteins (TJs) [8, 9]. Among TJs, claudin-5, occludin, and ZO-1 have been widely investigated and are regarded as essential factors for BBB integrity. We previously demonstrated that the breakdown of the BBB was attributed to the degradation of claudin-5 in early critical events in acute severe hypoglycemia, which result in edema formation [6, 10]. BBB breakdown is a particular characteristic of many neurological conditions, such as ischemic stroke, multiple sclerosis, and brain tumors [11]. Currently, many researchers are focusing on neurons and brain parenchyma, whereas straight BBB protection has attracted little attention. Therefore, targeting the structural changes in BMECs could prevent BBB breakdown and secondary neuronal injury [12].

Aquaporin-4 (AQP4) is the major water channel of the central nervous system, which is highly concentrated on the astrocyte endfeet and participates in the BBB functional unit [13, 14]. Several studies have shown the close association between AQP4 and the functions of the BBB [15, 16]. Their observations strongly demonstrated that AQP4 may serve as a pivotal molecule in the interactions between astrocytes and BMECs. However, how AQP4 regulates the BBB and TJs in the context of acute severe hypoglycemia remains unclear. Moreover, not only is it involved in water movement, but AQP4 may also contribute to cell adhesion and migration, which highlights the diversity and complexity of AQP4 functions [17–19]. Recently, the altered AQP4 expression has been considered to play a critical role in neuroinflammation during brain injury [20–22].

Neuroinflammation is an important common determinant of increased BBB permeability [23]. The nuclear peroxisome proliferator-activated receptors γ (PPAR-γ) are part of important signal transduction pathways that regulate inflammatory effects [24]. PPAR-γ activation and expression increase following brain damage and when inflammatory responses are developing. Once the PPAR-γ signaling pathway is activated, neuroinflammation is remarkably suppressed in many cerebral injuries. PPAR-γ present lower levels of expression in normal adult brains and are expressed in microglia and astrocytes, both of which are important cell types in neuroinflammation in response to neurological insults and brain damage [25, 26]. In astrocytes, PPAR-γ modulate the production of proinflammatory mediators, such as TNF-α, IL-1 β, and IL-6. Interestingly, PPAR-γ are also associated with edema [27]. Previous studies demonstrated that the activation of PPAR-γ can exert neuroprotective effects on many animal models of acute brain insults and can reduce brain edemas [28–30].

Our previous work has verified a rat model of hypoglycemia-induced brain edema and has shown that the breakdown of the BBB after hypoglycemia can be attributed to the degradation of TJs and altered AQP4 expression. Therefore, in this study, we demonstrated the neuroprotective effect of AQP4 deletion in a mouse model of severe hypoglycemia. Furthermore, we tried to identify possible new mechanisms of AQP4 deletion that are involved in the protection of BBB integrity by developing a model of hypoglycemia in AQP4 deletion mice. Moreover, these results will help in elucidating the role that astrocytes play in the pathogenesis of hypoglycemia.

## Methods

### Animals

Male AQP4^+/+^ and AQP4^−/−^ mice, aged 2 months old and weighing 22 to 25 g, were provided by Dr. Gang Hu, Jiangsu Key Laboratory of Neurodegeneration, Department of Anatomy, Histology, and Pharmacology of Nanjing Medical University in China. The mice were housed under environmentally controlled conditions with a 12-h light-dark cycle and a standard diet and water. All animals were treated according to the protocols approved by the Institutional Animal Care and Use Committee of Shanghai Jiao Tong University. Animals were deeply anesthetized with isoflurane prior to decapitation to minimize suffering.

### Induction of severe hypoglycemia in the mice

As previously described, severe hypoglycemia was induced by a single insulin injection, with some modifications [31]. An intraperitoneal insulin injection of 4 IU/kg regular insulin (Novolin-R, Novo Nordisk, Denmark) was administered to mice that had fasted for 12 h. The control group was injected with 0.9% saline (5 μL/g). Blood glucose levels were detected from the tail vein using an analyzer (Roche Performa). Blood glucose levels were measured prior to injection of insulin or saline, as well as 20, 40, 60, 80, 100, 120, and 140 min post-injection. Mice with blood glucose levels higher than 1.5 mmol/L at 40 and/or 60 min after the initial insulin injection received an additional dose of insulin (0.5 IU/kg). Mice were sacrificed following the blood glucose measurement at 2.5 h post-injection. The mice were separated into the following four groups: WT and AQP4^−/−^ mice in both normal and hypoglycemic conditions.

### Measurement of brain cortical water content

Mice from each of the four groups were anesthetized with isoflurane and sacrificed by decapitation. Each brain was divided into the two hemispheres via the midline. One half of the cortex was used for reverse transcription polymerase chain reaction (RT-PCR). The other half was used for water content measurement. The water content of the cortex, a sensitive indicator of cerebral edema, was evaluated to testify the presence of hypoglycemia-induced brain edema. Briefly, after decapitation, the brains were removed quickly, and the cerebellum and olfactory bulbs were discarded. Then, 100 mg samples of the brain tissue were immediately weighed to obtain the wet weight (W). The brain samples were then dried in an oven at 80 °C for 48 h and were then reweighed to obtain the dry weight (D). Brain water content (%) was calculated as (W − D) × 100/W.

### Evaluation of BBB permeability in vivo

The measurement of EB staining was determined using a method that was previously described [32]. Mice were injected with 2% EB (4 mL/kg; Sigma-Aldrich) dissolved in saline intravenously 1 h before decapitation. Under anesthesia, the mice were transcardially perfused with 60 mL of saline to remove the Evans blue from the vessels. Coronal sections were cut and photographed using a Cyber-shot camera (Sony, DSC-W390). In addition, each sample was immediately weighted, homogenized in 1 ml 50% trichloroacetic acid solution, and then centrifuged at 15,000*g* for 30 min. Then, the supernatant was diluted to 1:3 with ethanol, and its absorbance was measured at 610 nm using a Varioskan Flash Multimode Reader (Thermo Scientific). The amount of Evans blue dye was calculated using a standard curve and is expressed as microgram/gram brain tissue.

### RT-PCR quantification

Total RNA was extracted from the cortical brain tissue or cells using TRIzol reagent (Invitrogen), as previously described [6]. First-strand cDNAs were generated using an RT-PCR kit (K1622, Thermo Fisher Scientific). The primer sequences used for the RT-PCR were as follows: GAPDH sense 5′-TTCCTACCCCCAATGTATCCG-3′ and antisense 5′-C A T G A G G T C C A C C A C C C T G T T-3′; TNF-α sense 5′-C T T C T G T C T A C T G A A C T T C G G G G T-3′ and antisense 5′-A T C T G A G T G T G A G G G T C T G G G C-3′; IL-1β sense 5′- T G A C C T G T T C T T T G A G G C T G A C-3′ and antisense 5′- C A T C A T C C C A C G A G T C A C A G A G-3′; IL-6 sense 5′- G A A A T G A T G G A T G C T A C C A A A C T G-3′ and antisense 5′- G A C T C T G G C T T T G T C T T T C T T G T T-3′; and PPAR- γ sense 5′- G C T C C A A G A A T A C C A A A G T G C G-3′ and antisense 5′- G C T T C A A T C G G A T G G T T C T T C G-3′. SYBR Green mix (F-415XL, Thermo Fisher Scientific) was used to perform quantitative-PCR (qPCR) reactions (7300, Applied Biosystems). GAPDH was used as the internal control. Each sample was run in triplicate. The expression of each gene of interest in other groups compared to the WT was calculated using the 2^−ΔΔC^_*t*_ method.

### Western blotting

Western blotting was performed as in our previous report [10]. Tissues and cells were homogenized with a lysis buffer containing radioimmunoprecipitation assay buffer (Thermo Fisher Scientific) and protease inhibitor cocktails on ice. Samples containing equal amounts of total protein were administered 8 or 12% acrylamide denaturing gels (SDS-PAGE) before being transferred onto the nitrocellulose membranes. After being blocked with 5% skimmed milk for 1 h, the membranes were successively incubated with antibodies against AQP4 (1:400, Santa Cruz Biotechnology), GFAP (1:500, sigma), PPAR-γ, and claudin-5 (1:500, Invitrogen), which were followed by incubation with secondary antibodies (anti-rabbit IRDye-700 nm or anti-mouse IRDye-800 nm) (Roche). β-actin served as the internal control. After being washed with TBST, the membranes were visualized and analyzed using an Odyssey imaging system (LI-COR Bioscience, Lincoln, NE, USA). Western blots were repeated at least three times for each sample.

### Immunofluorescence staining

The mice were perfused transcardially with precooled PBS and 4% paraformaldehyde (pH 7.4; Sigma) in deep anesthesia. The brain samples were removed and immersed in the same fixative overnight and then were placed in graded sucrose solutions (10, 20, or 30%) for dehydration. Frozen sections (20 μm) were immersed in 0.3% Triton X-100 for permeabilization, were blocked with 10% goat serum, and then were incubated with a mixture of primary antibodies for AQP4 (1:200, Santa Cruz Biotechnology) and claudin-5 (1:200, Invitrogen) overnight at 4 °C and subsequently with a mixture of two secondary antibodies, namely, FITC 488-conjugated goat anti-rabbit and DyLight 594-conjugated goat anti-mouse (1:1000, Jackson) for 1 h at room temperature. Nuclei were stained with 4′,6-diamidino-2-phenylindole (DAPI). The slices were then mounted with coverslips using antifade reagent (Molecular Probes, Invitrogen) and were sealed with nail polish. Controls were made by omitting primary antibodies from the incubation process. Images were acquired with a laser confocal microscope (SP8; Leica Microsystems).

Cell cultures were fixed and permeabilized with 0.2% Triton X-100 for 20 min then were blocked with 5% goat serum for 30 min. Subsequently, the slides were incubated with primary antibodies against GFAP (1:200, Sigma) and AQP4 (1:400) for 2 h at room temperature then were incubated with DyLight 594-conjugated goat anti-mouse (1:1000) for 1 h at room temperature. Images were acquired with the same laser confocal microscope.

### AQP4 siRNA knockdown in the astrocytes

Three different sequences of siRNA (si-AQP4-1, si-AQP4-2, and si-AQP4-3) targeting AQP4 were used to suppress AQP4 protein in astrocytes. The isolation of primary astrocytes was performed as described previously [6]. To test the knockdown efficiency, astrocytes cultured until 70% confluence were seeded in 6-well plates and were transfected with three AQP4 siRNAs (5 μg/500 μL Opti-MEM) or control siRNA (si-Sc) (5 μg/500 μL Opti-MEM) with two doses of Lipofectamine 3000 reagent (3.75 μL or 7.5 μL/500 μL Opti-MEM) (Thermo Fisher Scientific), in accordance with the manufacturer’s instructions. Thus, the astrocytes were separated into the following groups: control, si-Sc-1, si-Sc-2, si-AQP4-1-1, si-AQP4-1-2, si-AQP4-2-1, si-AQP4-2-2, si-AQP4-3-1, and si-AQP4-3-2. After being transfected for 48 h, all the groups were stimulated with normal glucose medium (5.5 mM) and glucose deprivation (GD, 0 mM), and then the total proteins were collected after 24 h for Western blot analysis to assess the silencing effect of the different types of siRNA. The most efficient group was then chosen to be used in the following experiment. The following AQP4 siRNAs were used: AQP4 siRNA 1 (sense, 5′-G A U C A G C A U C G C C A A G U C U T T-3′; antisense, 5′-A G A C U U G G C G A U G C U G A U C T T-3′); AQP4 siRNA 2 (sense, 5′-A G A C U U G G C G A U G C U G A U C T T-3′; antisense, 5′-G A U C A G C A U C G C C A A G U C U T T-3′); AQP4 siRNA 3 (sense, 5′-G A U C C U C U A C C U G G U C A C A T T-3′; antisense, 5′-U G U G A C C A G G U A G A G G A U C T T-3′); and negative control siRNA (siSc) (sense, 5′-G G C U C U A G A A A A G C C U A U G C T T-3′; antisense, 5′-G C A U A G G C U U U U C U A G A G C C T T-3′) (GenePharma, Shanghai, China).

### Non-contact astrocyte-bEnd.3 co-culture and treatment

Immortalized mouse bEnd.3 cells were purchased from the American Type Culture Collection. Given that the non-contact astrocyte-bEnd.3 co-culture model represents one of the better in vitro models of the human BBB, this particular model was used for this study. Primary astrocytes and bEnd.3 cells were cultured separately, as previously reported [6, 10]. To produce an in vitro model of the BBB, bEnd.3 cells (4 × 10^4^ cells/cm^2^) were seeded on the inner surface of transwell inserts (0.4 μm pore size, Corning Costar) in a 12-well tissue culture plate. The bEnd.3 cells were grown for 1 day. Astrocytes (2 × 10^4^ cells/cm^2^) were seeded on the inside of the12-well culture plates (Costar), and then siRNA of AQP4 was added, followed by 1 day of incubation. Then, the transwell inserts were placed into the 12-well plates containing confluent layers of astrocytes with AQP4 knockdown (siAQP4) or without AQP4 knockdown (control). Fresh medium (0.5 mL within the insert and 1.5 mL in the outer well) was provided every other day. For the hypoglycemic conditions, co-cultures of bEnd.3 cells and AST^+/+^ or AST^−/−^ were subjected to 5.5 and 0 mM glucose for 24 h. Thus, the co-culture models were separated into four groups: control + 5.5 mM glucose, control + 0 mM glucose, siAQP4 + 5.5 mM glucose, and siAQP4 + 0 mM glucose. For the condition of PPAR-γ treatment, co-culture models were cultured in normal growth medium for 3 days, followed by culturing in normal glucose (5.5 mM), GD (0 mM), or GD with 10 μM GW9662 (an antagonist of PPAR-γ) for 24 h. Protein from the bEnd.3 cells was harvested for Western blot examination.

### Evaluation of BBB permeability in vitro

The integrity and function of the non-contact co-culture BBB models were studied using assessments of paracellular flux of sodium fluorescein (NaF). Evaluation of BBB permeability in vitro was performed as described in our previous report [10]. All experiments were performed at least in triplicate.

### Enzyme-linked immunosorbent assay

Brain tissues were harvested from mice after hypoglycemic stress. Supernatants from the cerebral cortex were analyzed for cytokines. The production of cytokines by astrocytes is of particular interest because astrocytic involvement in the regulation of CNS inflammation is complex. To explore how reactive astrocytes may participate in BBB breakdown induced by hypoglycemia, we detected the protein levels of IL-1β, IL-6, and TNF-α and quantitated these levels using a specific ELISA kit (BioLegend), following the manufacturer’s instructions. Briefly, the supernatants were collected from the co-culture media, were subjected to various experimental conditions, and were centrifuged at 3000*g* for 10 min at 4 °C to remove the debris. Standards or samples were then added to 96-well plates pre-coated with different capture antibodies and were then incubated at room temperature for 2 h. Then, the plates were washed with PBST. Each assay was tested in triplicate.

### Statistical analysis

The statistical analyses were performed using the SPSS version 18.0 software for Windows (SPSS Inc., Chicago, IL, USA). The results are presented as the mean ± standard deviation. One-way analysis of variance (ANOVA) followed by the post hoc Dunnett’s test was used to compare differences between the mean values of data involving more than two groups. Differences with *p* values < 0.05 were considered significant.

## Results

### AQP4 deletion attenuated early brain edema during hypoglycemic insult

**Fig. 1:**
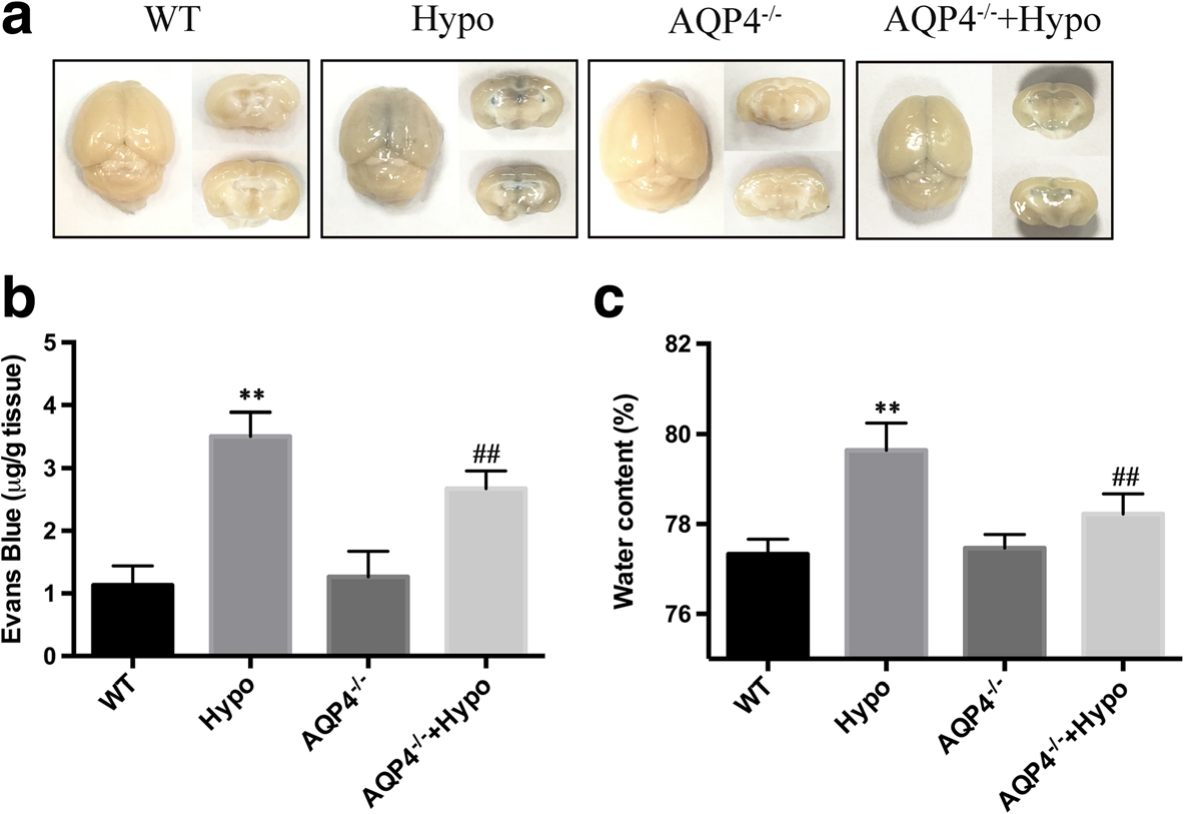
AQP4 deletion attenuated hypoglycemia-induced brain edema. **a** Representative photographs of the whole EB-stained brain. Each image shown is representative of five independent mice. **b** Quantification of the EB extravasation. **c** Water content was calculated by (W − D)/W × 100%. *n* = 5 mice/group. **p < 0.01 vs. the WT group, ^##^*p* < 0.01 vs. the Hypo group

Increased BBB permeability during severe hypoglycemia has been shown in our previous reports [6, 10]. To test whether AQP4 deficiency attenuated brain edema under acute hypoglycemic conditions, we quantified the extravasation of EB and brain water content in mice under severe hypoglycemia (Fig. 1a). The brains from the WT groups were barely stained with EB, and the brains from the Hypo group were obviously stained with EB. As quantified in Fig. 1b, there was background EB dye detected in the WT group. However, hypoglycemia induced significant increases in the extravasation of EB, and AQP4 deletion significantly decreased EB leakage (2.67 ± 0.28 vs. 3.51 ± 0.38 μg/g brain weight, *p* < 0.01), suggesting that AQP4 preserved the alteration of BBB permeability under acute hypoglycemic conditions. We further calculated the amount of water in the brain by weighing the brain tissue before and after drying. The results showed that acute severe hypoglycemia induced significant cerebral edema in the Hypo group, as demonstrated by an average of 3% of increased water compared with the WT group. However, AQP4 deletion significantly reduced the increased water ratio in the hypoglycemia model by approximately 30% (Fig. 1c) in the AQP4^−/−^ + Hypo group. This analysis showed that AQP4 deletion decreased BBB permeability in the presence of hypoglycemia.

### AQP4 deletion attenuated hypoglycemia-induced BBB permeability

**Fig. 2:**
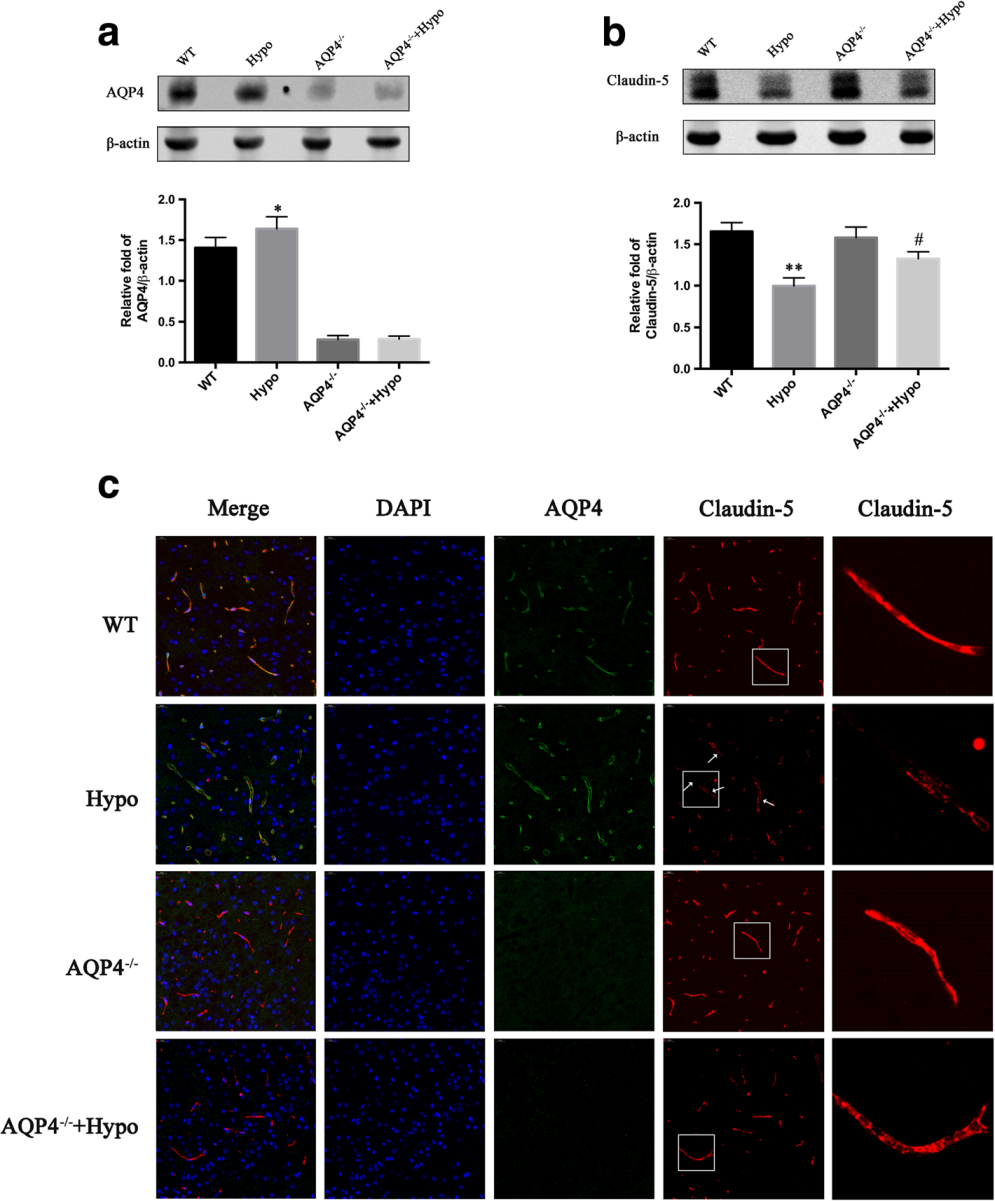
AQP4 deletion attenuated hypoglycemia-induced BBB disruption. **a** Western blot analysis revealed a slight increase in AQP4 in the Hypo compared to the WT. **b** A significant increase in claudin-5 expression in the AQP4^−/−^ + Hypo was found with hypoglycemic edema compared to the Hypo. **c** The sections of the brain cortex were co-stained for AQP4 (green) and claudin-5 (red). Nuclei were stained with DAPI. Confocal microscopic images showed continuous and linear labeling of claudin-5 along the vessels in the WT group and the AQP4^−/−^ group. In contrast, discontinuous labeling and gap formation were observed in the Hypo group (white arrows). AQP4 deletion significantly reduced gap formation and maintained the integrity of claudin-5. The images in the square frames of the graphs were amplified as images. Scale bar = 20 μm. *n* = 5 per group, *p < 0.05 or **p < 0.01 vs. the WT group, ^#^*p* < 0.05 vs. the Hypo group

It was previously shown that increased AQP4 and degradation of claudin-5 were involved in acute hypoglycemia-induced brain edema both in vivo and in vitro [6, 10]. However, nothing is known about the effect of AQP4 deletion on BBB disruption induced by hypoglycemic stress. Here, in order to investigate the role of AQP4 deficiency in hypoglycemia-induced degradation of BBB components, we analyzed the expression of claudin-5, a major tight junction protein, and AQP4 via Western blot. We also examined the localization of claudin-5 and AQP4 in the brain cortex of the brain using claudin-5/AQP4 double staining. The Western blot results showed that claudin-5 expression was downregulated after hypoglycemia. Interestingly, hypoglycemia-induced claudin-5 downregulation was alleviated in the AQP4^−/−^ + Hypo mice. (*p < 0.05 and **p < 0.05 vs. WT group). However, AQP4 deletion preserved the loss of claudin-5 caused by hypoglycemia (^#^*p* < 0.05 vs. Hypo group) (Fig. 2b). Furthermore, immunofluorescence staining for AQP4 (green) and claudin-5 (red) was performed in the cortex of the brain after hypoglycemia in mice (Fig. 2c). The results of the immunofluorescence analysis showed that the astrocytic endfeet marker AQP4 was expressed at high levels in the Hypo mice. Confocal microscopy analysis showed that claudin-5-positive staining was continuously located on the endothelial cell margin of the cerebral microvessels in the WT mice; this continuity was disrupted by hypoglycemia. It was noted that this process was reversed, and gap formation was greatly reduced in the AQP4^−/−^ + Hypo mice.

### AQP4 deletion decreased inflammatory responses by upregulating the PPAR-γ signaling pathways during hypoglycemic stress

**Fig. 3:**
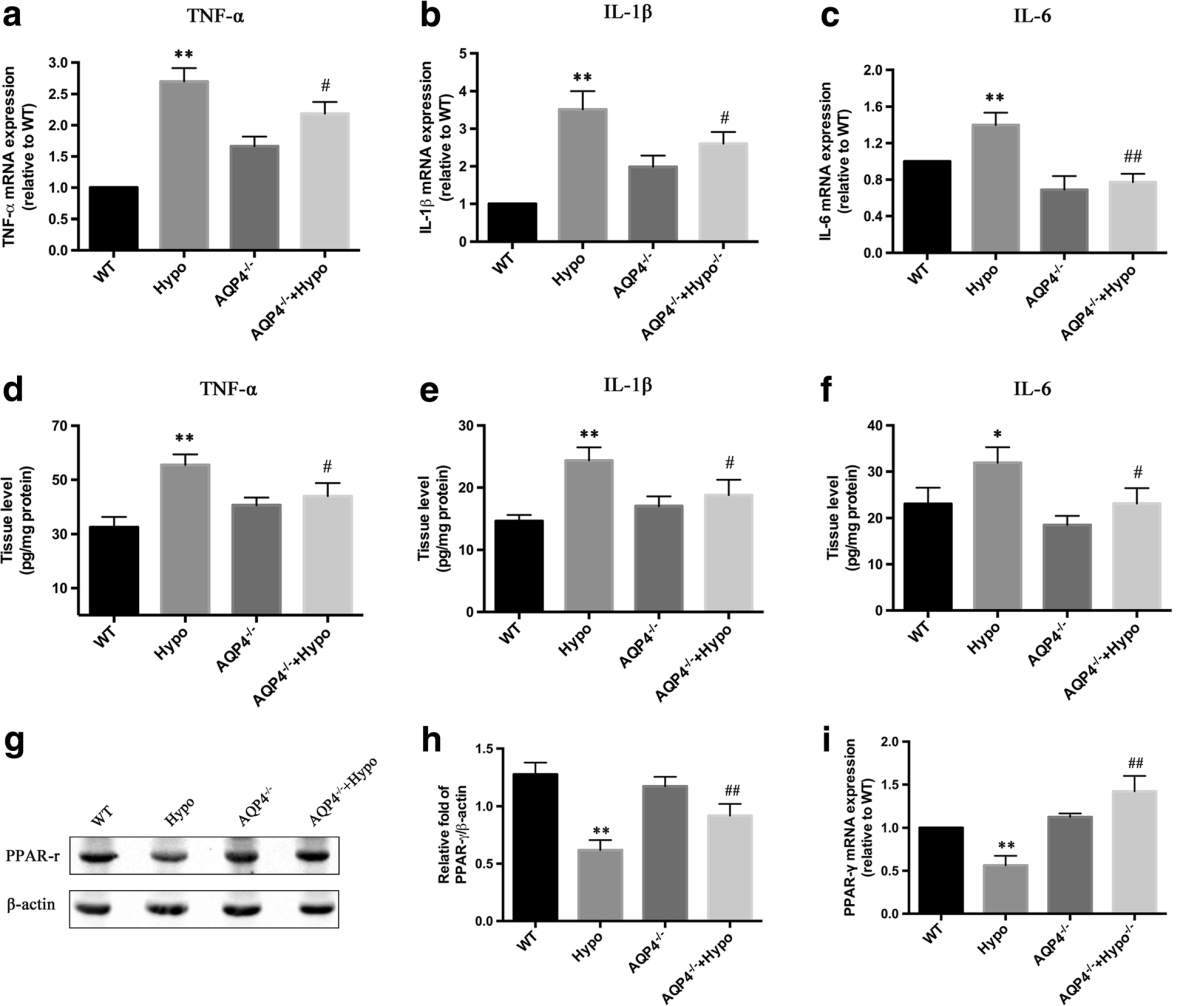
AQP4 deletion decreased inflammatory responses by upregulating the PPAR-γ signaling pathways during hypoglycemic stress. **a** The fold change of mRNA expression of TNF-α. **b** The fold change of mRNA expression of IL-1β. **c** The fold change of mRNA expression of IL-6. The expression levels and mRNA of TNF-α, IL-1β, and IL-6 were significantly increased in the Hypo group compared to the WT group; however, they were significantly reduced in the AQP4^−/−^ + Hypo group compared to the Hypo group. **d** ELISA analysis of TNF-α proteins in the cerebral cortex from mice. **e** ELISA analysis of IL-1β proteins in the cerebral cortex from mice. **f** ELISA analysis of IL-6 proteins in the cerebral cortex from mice. **g** Western blot analysis of PPAR-γ protein expression. **h** Quantitative analysis of PPAR-γ protein expression. **i** The mRNA expression of PPAR-γ in the brains of mice subjected to hypoglycemia. *P < 0.05 or **P < 0.01 vs. the WT group, ^#^*p* < 0.05 or ^##^*p* < 0.01 vs. the Hypo group. *n* = 3 in each group

Inflammation plays a very important role in BBB dysfunction. Hypoglycemia can evoke a strong inflammatory response characterized by activated inflammation-related signaling pathways, leading to increased expression of inflammation-related genes and, ultimately, to the release of proinflammatory factors that exacerbate BBB damage. To determine whether the effect of AQP4 deletion on BBB disruption after hypoglycemia is involved in the immunomodulatory influence of AQP4 deletion, we examined TNF-α, IL-1β, and IL-6 mRNA expression in the brains of mice (Fig. 3a–c). We demonstrated that the expression of TNF-α, IL-1β, and IL-6 was increased following acute severe hypoglycemia. The three cytokines were significantly decreased in the AQP4^−/−^ + Hypo group compared to the Hypo group. There was also a significant decrease in the protein levels of TNF-α, IL-1β, and IL-6 in the AQP4^−/−^ + Hypo group compared to the Hypo group (Fig. 3d–f). It was demonstrated that AQP4 deletion decreased the production of TNF-α, IL-1β, and IL-6 in the hypoglycemic stress. Recent studies have shown that PPAR-γ is an instrumental pleiotropic regulator of anti-inflammation, and anti-oxidative regulation negatively regulates gene expression of proinflammatory proteins. Therefore, we hypothesized that the suppression of inflammation in AQP4^−/−^ mice subjected to hypoglycemia is associated with dysregulation of PPAR-γ. As shown in Fig. 3g, h, hypoglycemic stress significantly decreased the expression level of PPAR-γ in the Hypo mice, which was also increased in the AQP4^−/−^ + Hypo mice. Moreover, hypoglycemic stress also decreased the mRNA levels of PPAR-γ in the Hypo mice (Fig. 3i), but these levels dropped dramatically in the AQP4^−/−^ + Hypo mice in response to hypoglycemic stress.

### Hypoglycemic stress increased GFAP in astrocytes with AQP4 knockdown or without AQP4 knockdown

**Fig. 4:**
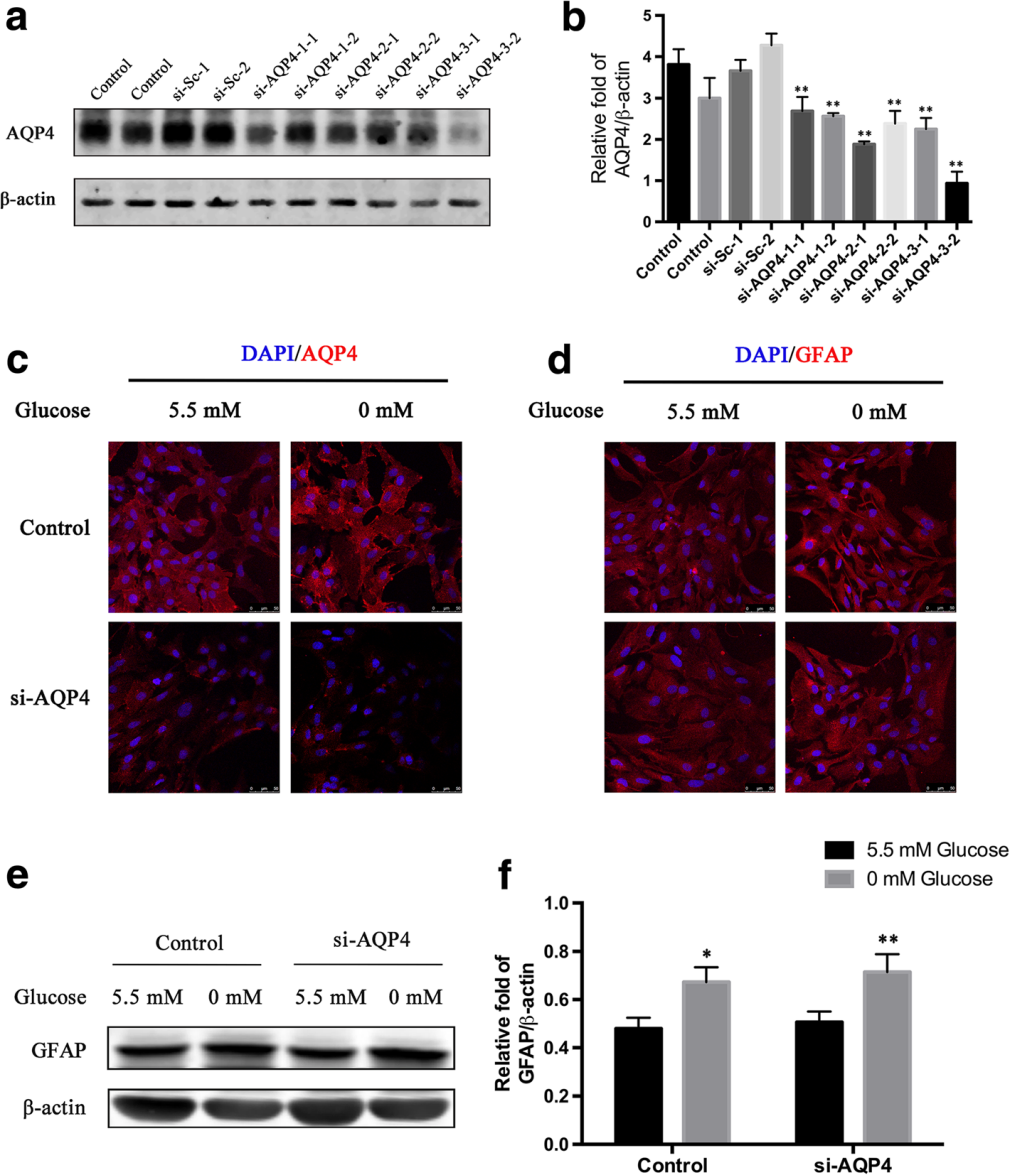
GD induced high expression of GFAP in the astrocytes. Astrocytes were transfected with siRNA against AQP4, and Western blot analysis was used to detect the efficiency of the different siRNA sequences. **a** The knockdown efficiency of the three siRNA sequences targeting AQP4, as determined by Western blot analysis. **b** Quantification of AQP4 expression. **p < 0.01 vs. the control or si-Sc group. **c** Astrocytes exposed to GD for 24 h showed selective staining of AQP4 on the plasma membrane. **d** GFAP was overexpressed in astrocytes subjected to GD with or without AQP4 knockdown, while no alterations were found in the astrocytes with AQP4 knockdown compared to the control. **e** The levels of GFAP protein in astrocytes were detected via Western blot. **f** Quantification of GFAP expression. *p < 0.05 or **p < 0.01 vs. the control + 5.5 mM glucose group. *n* = 3 in each group

Despite all the evidence given by the animal studies abovementioned, we also aimed to clarify the specific involvement of AQP4, which is expressed mainly by astrocytes. We decided to use primary astrocytes to dissect the specific role played by AQP4 under GD exposure and, thus, to complement the animal studies. In vitro cultured astrocytes were identified by staining with GFAP and DAPI (Additional file 1: Figure S1). Furthermore, we performed knockdown experiments via transfection of astrocytes with siRNA against AQP4 (si-AQP4). The knockdown efficiency was confirmed via Western blot (Fig. 4a). The results showed that AQP4 expression in the astrocytes was significantly decreased, and si-AQP4-3-2 was the most efficient siRNA sequence; this sequence suppressed AQP4 protein expression by 80%, while the expression was not affected by the control or si-Sc (Fig. 4b). Therefore, we used the si-AQP4-3-2 group for the following experiments.

To further elucidate the effect of AQP4 deletion on astrocytes, immunofluorescence was performed to stain AQP4 and GFAP proteins. The results showed that the cellular distribution of AQP4 and the astrocytic morphology alterations were induced by 0 mM glucose medium. There was a clear increase in AQP4 expression in the membrane. Therefore, we can conclude that there was a shift from cytoplasmic localization to membrane localization during hypoglycemia (Fig. 4c). GFAP protein levels in the astrocytes were also evaluated using immunofluorescence, and AQP4 knockdown was found to have no effect on GFAP protein levels, but GFAP levels increased in the astrocytes exposed to GD in absence/presence of AQP4 (Fig. 4d). The protein extracted from astrocytes was subjected to Western blot analysis using anti-GFAP antibodies. The expression of GFAP was increased in the astrocytes exposed to GD with AQP4 knockdown or without AQP4 knockdown (Fig. 4e, f).

### AQP4 knockdown in astrocytes protects against hypoglycemia-induced BBB permeability

**Fig. 5:**
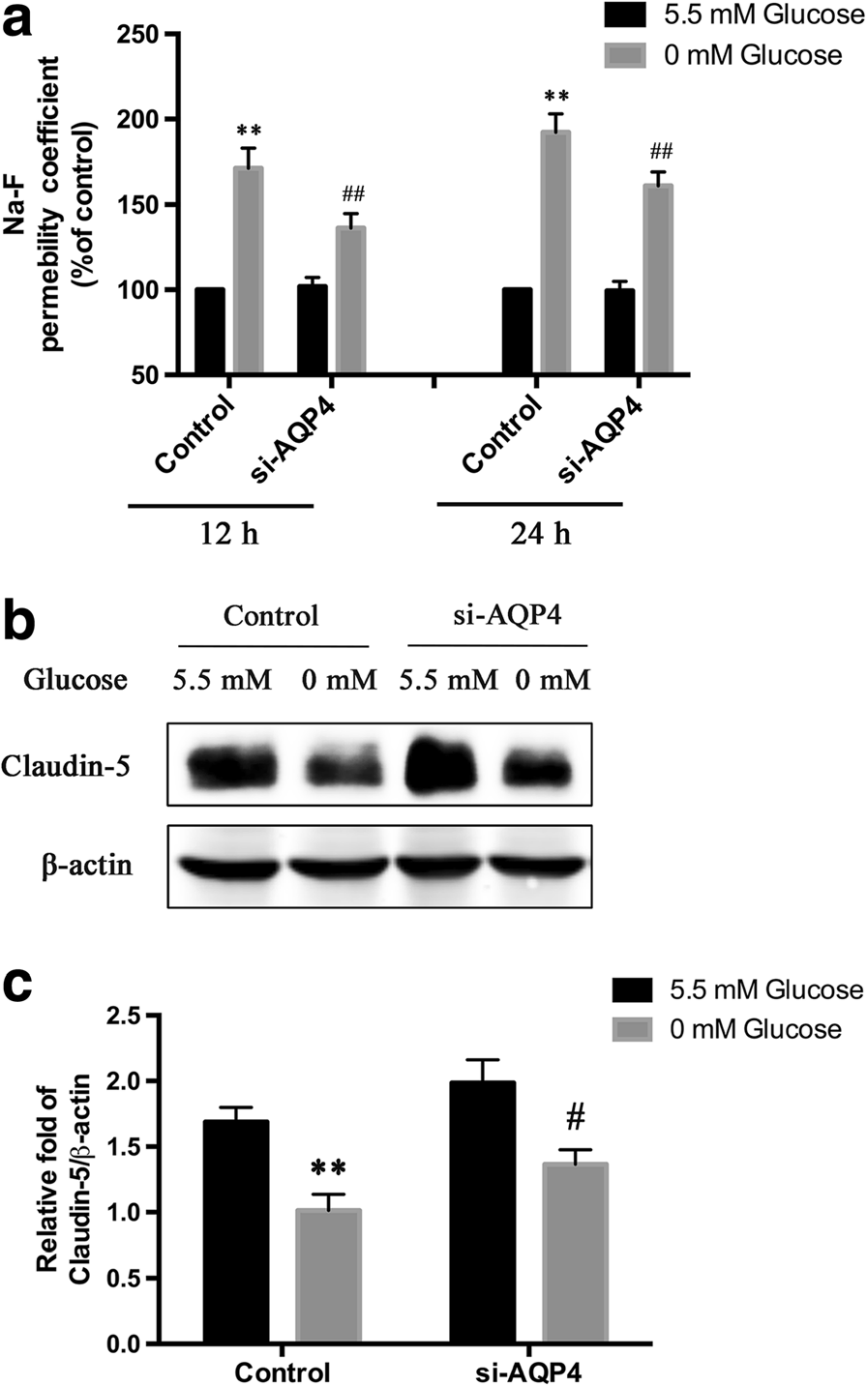
AQP4 knockdown ameliorated GD-induced disruption of in vitro BBB models. **a** The effects of AQP4 knockdown on in vitro BBB permeability after GD. **b** The levels of claudin-5 protein in bEnd.3 cells were detected via Western blot. **c** Quantification of claudin-5 expression. **p < 0.01 vs. the control + 5.5 mM glucose group, ^#^*p* < 0.05 or ^##^*p* < 0.01 vs. the control + 0 mM glucose group. *n* = 3 in each group

To determine whether the knockdown of AQP4 in astrocytes was capable of preventing BBB disruption triggered by hypoglycemia in an indirect way, we set up non-contact co-cultures of astrocytes without AQP4 and bEnd.3 cells and assessed transendothelial permeability by measuring the transendothelial transport of Na-F 12 or 24 h after treatment with 0 mM glucose. The results showed that exposing the control group to 12 and 24 h of 0 mM glucose medium increased transendothelial permeability by a decrease of approximately 71 and 92%, respectively, compared to that of 5.5 mM glucose medium (Fig. 5a). In order to exclude the direct effects of GD on bEnd.3, we also detected the effects of GD on the transendothelial permeability of monoculture model (only bEnd.3) and co-culture model (non-contact astrocyte-bEnd.3 co-culture). As shown in Additional file 2: Figure S2A, the differences of transendothelial permeability between the monoculture group and co-culture group were not statistically significant at 12 and 24 h. AQP4 knockdown significantly decreased permeability by approximately 35 and 31% at 12 and 24 h, respectively, compared with that in the control +  0 mM glucose group. To determine whether the AQP4-induced regulation of BBB permeability was associated with an increase in tight junctions, we detected claudin-5 via Western blot (Fig. 5b). The results showed that the expression of claudin-5 was downregulated in the control + 0 mM glucose group compared to that in the control + 5.5 mM glucose group at 24 h. AQP4 knockdown increased the expression of claudin-5 in the si-AQP4 + 0 mM glucose group compared to that in the control + 0 mM glucose group (Fig. 5c). Thus, the knockdown of AQP4 significantly decreased hypoglycemia-induced high transendothelial permeability, indicating a protective role of AQP4 in a hypoglycemic in vitro BBB model.

### PPAR-γ was involved in the protective effect of AQP4 knockdown on BBB integrity via anti-inflammation

**Fig. 6:**
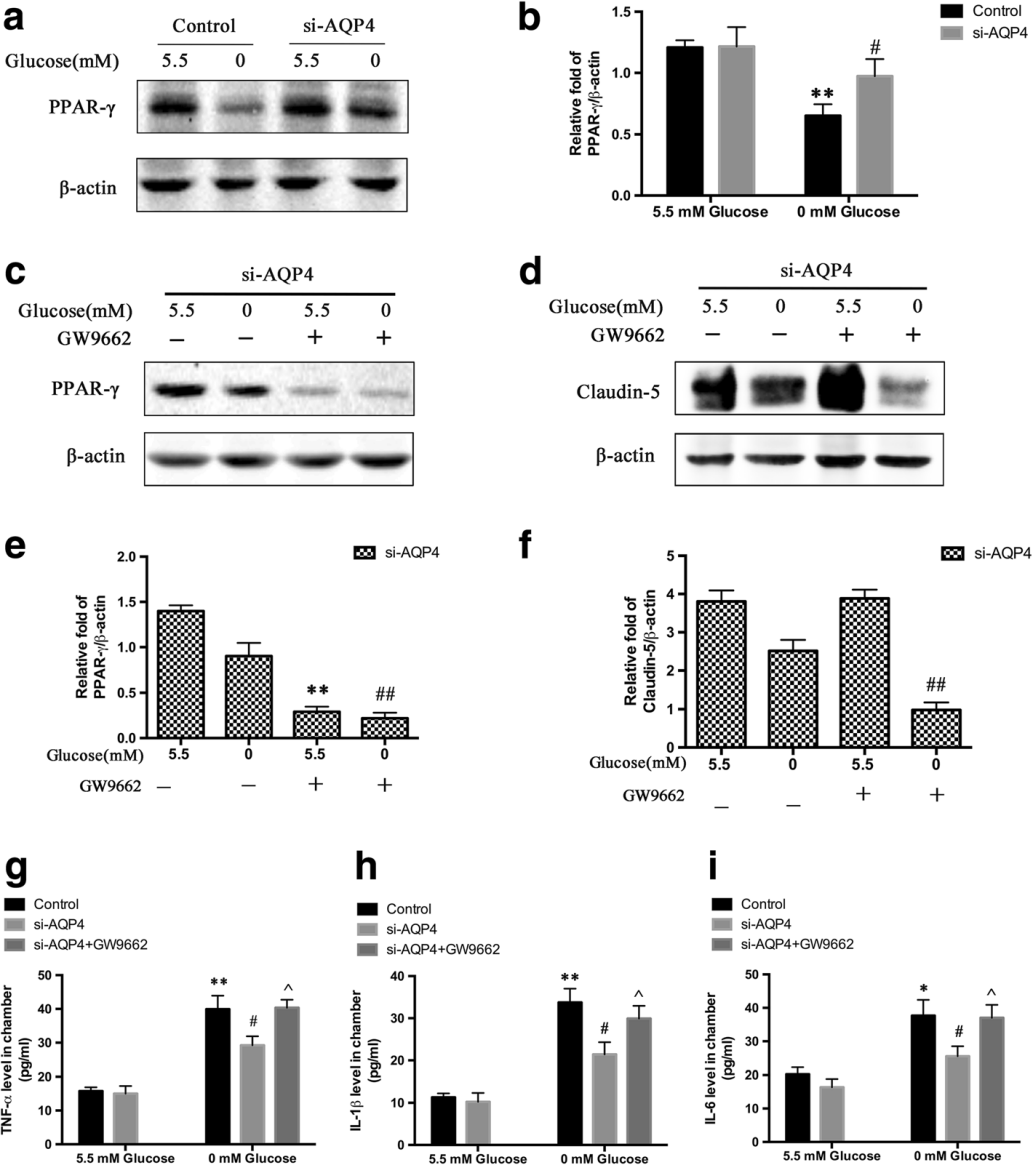
PPAR-γ was involved in the protective effect of AQP4 knockdown on BBB integrity via anti-inflammation. **a** PPAR-γ expression in astrocytes was quantified via Western blot. **b** Quantification of PPAR-γ. **p < 0.01 vs. the control + 5.5 mM glucose group, ^#^*p* < 0.05 vs. the control + 0 mM glucose group. **c** The validity of the inhibitor GW9662 against PPAR-γ was confirmed via Western blot. **d** GW9662 significantly reduced claudin-5 expression in the co-culture models. **e** Quantification of PPAR-γ expression. **p < 0.01 vs. the si-AQP4 + 5.5 mM glucose group, ^##^*p* < 0.01 vs. the si-AQP4 + 0 mM glucose group. **f** Quantification of claudin-5 expression. ^##^*p* < 0.01 vs. the si-AQP4 + 0 mM glucose group. **g** TNF-α levels in the co-culture media from astrocytes as measured by ELISA. **h** IL-1β levels in the co-culture media from astrocytes as measured by ELISA. **i** IL-6 levels in the co-culture media from astrocytes as measured by ELISA. *p < 0.05 or **p < 0.01 vs. the control + 5.5 mM glucose group, ^#^*p* < 0.05 vs. the control + 0 mM glucose group, ^^^*p* < 0.05 vs. the si-AQP4 + 0 mM glucose group. *n* = 3 in each group

To study the potential mechanism of the protective effect of AQP4 knockdown in astrocytes on BBB integrity in response to hypoglycemic stress, the level of PPAR-γ in astrocytes was examined using Western blot analysis (Fig. 6a). We found that the expression of PPAR-γ in cultured astrocytes during GD was significantly enhanced in response to AQP4 knockdown. To further elucidate whether the PPAR-γ pathways were involved in the protective effect of AQP4 knockdown, we detected the effect of a PPAR-γ inhibitor (GW9662, Selleck Chemicals) on claudin-5 expression. The validity of the inhibitor was confirmed via Western blot (Fig. 6c). As shown in Fig. 6d, GW9662 significantly reduced claudin-5 expression. In order to exclude the direct effects of PPAR-γ inhibition on bEnd.3, further study showed that the difference of claudin-5 expression between GD without GW9662 and GD with GW9662 was not statistically significant. It was demonstrated that the direct effects of PPAR-γ inhibition on bEnd.3 were not significant when bEnd.3 were exposed to GD (Additional file 2: Figure S2B, C). The aforementioned data showed that AQP4 deletion decreased inflammatory responses by upregulating the PPAR-γ signaling pathways in the mouse hypoglycemia model. However, it is unknown whether the changes in the levels of TNF-α, IL-1β, and IL-6 were induced by astrocytes and whether the protective effect of AQP4 knockdown could profoundly contribute to the activation of PPAR-γ. We then detected the levels of TNF-α (Fig. 6g), IL-1β (Fig. 6h), and IL-6 (Fig. 6i) derived from astrocytes via ELISA. The ELISA assay revealed that the levels of TNF-α, IL-1β, and IL-6 in the media of cultured astrocytes were significantly increased in the control + 0 mM glucose group compared with those of the si-AQP4 + 5.5 mM glucose group. Moreover, the high levels of TNF-α, IL-1β, and IL-6 were suppressed by AQP4 knockdown. However, the suppression of inflammation was abrogated in response to GW9662 in the si-AQP4 + 0 mM glucose group. These in vitro findings also indicated that AQP4 knockdown suppressed inflammatory activities via the PPAR-γ pathway in cultured astrocytes in order to exert its neuroprotective function.

TNF-α, IL-1β, and IL-6 can be produced and released by astrocytes in an autocrine manner [33]. These results demonstrated that AQP4 knockdown has the ability to activate PPAR-γ proteins, an ability that was inhibited by GW9662. However, the expression of the important inflammatory cytokines, IL-1β, TNF-α, and IL-6, was remarkably attenuated in the si-AQP4 + 0 mM glucose group compared to that in the control + 0 mM glucose group, but the expression of these cytokines was elevated again rapidly after treatment with GW9662. These data suggested that AQP4 knockdown can activate PPAR-γ, which contributed to the anti-inflammatory effects during hypoglycemic stress.

## Discussion

The BBB is damaged in an early critical event in response to severe hypoglycemia, which causes an inflammatory cascade, edema formation, and, ultimately, serious outcomes [34–36]. BBB disruption directly resulting from vascular endothelial damage potentially aggravates vasogenic edema and worsens prognosis. Thus, maintaining BBB integrity is critical for reducing secondary brain injury following hypoglycemia. There is increasing evidence that acute hypoglycemia induces a proinflammatory environment that may contribute to vascular injury. Proinflammatory cytokines, such as TNF-α, IL-1β, and IL-6, have been found to be capable of disrupting the epithelial barrier by decreasing tight junction protein expression [37, 38]. Despite it being well-documented that AQP4 is implicated in proinflammatory features of astrocytes [39, 40], little is known about whether astrocytes with AQP4 deficiencies may contribute to the protective effect of alleviating BBB disruption by reducing inflammatory responses under hypoglycemic stress. In this study, in vivo experiments demonstrated that AQP4^−/−^ mice exhibit a protection against BBB disruption with reduced brain edema compared to the WT controls in response to an acute severe hypoglycemic condition. The in vitro results supported that this was due to an attenuated inflammatory response that was due to the activation of the PPAR-γ pathway in astrocytes. Thus, we insisted that the effect of AQP4 deletion on ameliorating BBB disruption was due to the suppression of proinflammatory cytokine production, which resulted in less degradation of tight junction proteins.

It is known that AQP4-deletion animals do not show any differences when compared to wild-type animals regarding brain water content, neurological status, BBB properties, and pericapillary astrocytic foot process areas under normal conditions [41]. However, it was unclear whether there is a link between AQP4 and BBB integrity under hypoglycemic conditions. In accordance with our results, others have shown that AQP4 inhibition prevents edema formation and BBB disruption under the condition of meth use [42, 43]. To better confirm the effects of AQP4, we used AQP4^−/−^ mice. We found that AQP4 deletion reduced brain edema and BBB permeability under hypoglycemic stress. AQP4 deletion attenuated BBB disruption after hypoglycemia, but injuries in the AQP4^−/−^ + Hypo mice were still severe. In addition, as TJ has essential roles in barrier functions and consist of integral membrane proteins, we analyzed three important TJ proteins: occludin, ZO-1, and claudin-5 in preliminary experiments. The results showed that AQP4 deletion upregulated claudin-5 compared to occludin and ZO-1 (the data are not shown). Thus, we put emphasis on claudin-5.

It is well known that hypoglycemia-induced release of proinflammatory factors, including TNF-α, IL-1β, and IL-6 [7, 44], plays a critical role in BBB damage. To investigate whether these factors may contribute to a depressed effect of AQP4 deletion on BBB integrity, we evaluated their mRNA expression profiles and protein levels. Our observations indicated that the levels of TNF-α, IL-1β, and IL-6 were upregulated under hypoglycemic stress; in addition, the protein levels of PPAR-γ were decreased under hypoglycemic condition. However, AQP4 deletion was able to suppress the high levels of TNF-α, IL-1β, and IL-6 and activate the PPAR-γ pathway under hypoglycemic stress. These in vivo findings suggested that AQP4 deletion decreased inflammatory responses by upregulating the PPAR-γ signaling pathways during hypoglycemic stress. We further investigated the mechanisms involved in order to determine whether AQP4 deletion elicited its protective effects by suppressing inflammatory responses.

It is noted that astrocytes contribute to BBB formation by inducing the BBB phenotypic characteristics of endothelial cells. Furthermore, astrocytes also have shown a capability to promote tight junction formation in the brain’s capillary endothelium. Previous studies revealed reduced cytokine release by astrocytic cultures in conditions of AQP4 deficiency [39]. To investigate whether the decrease in cytokine secretion by astrocytes is related to AQP4 deficiency, we measured the secretion of TNF-α, IL-1β, and IL-6 in AQP4-knockdown astrocytes subjected to GD. The results showed significantly decreased TNF-α, IL-1β, and IL-6 secretion at 24 h after GD in the AQP4-knockdown astrocytes compared to astrocytes, a finding that was consistent with those of previous reports [34].

Activation of PPAR-γ is thought to prevent claudin-5 downregulation in the lungs of HIV-infected animals [45]. Growing evidence has indicated that PPAR-γ activity modulates immune inflammatory responses [46, 47]. Thus, we hypothesized that dysregulation of PPAR-γ is involved in vascular injury induced by hypoglycemia. Our data showed significant downregulation of PPAR-γ transcription and expression in the Hypo group. This suggested that suppression of PPAR-γ expression may be involved in the high levels of TNF-α, IL-1β, and IL-6 induced by hypoglycemia. Studies using in vitro-cultured astrocytes have shown that glucose deprivation also suppresses PPAR-γ. However, AQP4 deficiency can reverse this phenomenon. The in vivo and in vitro results showed that AQP4 deficiency reversed the downregulation of PPAR-γ under hypoglycemic stress. This suggested that PPAR-γ activation prevents hypoglycemia-induced downregulation of claudin-5 both in vitro and in vivo. To confirm the results showing that PPAR-γ activation in astrocytes with AQP4 deficiency diminished GD-induced downregulation of claudin-5, we added GW9962, an inhibitor of PPAR-γ, to the si-AQP4 co-culture group. The production of lactate in astrocyte cultures has been reported [48]. In order to contribute to the elucidation of the function of strocytic gluconeogenesis, a supplemental examination has been made to detect the amount of lactate in the astrocyte culture medium. There was a significant increase in the levels of lactate in GD conditions (Additional file 3: Figure S3). However, AQP4 deletion did not affect the energy supply to bEnd.3 in the GD conditions. Studies are still ongoing in our laboratory to further explore these pathways and to determine the mechanisms through which PPAR-γ activation exerts its protective effects against hypoglycemia-induced brain injury.

## Conclusions

In summary, our present study showed that AQP4 deficiency exerts a potent regulatory function on PPAR-γ expression in astrocytes, which, consequently, attenuates proinflammatory responses, hypoglycemia-induced BBB permeability, and brain edema. Based on these findings, we believe that the effect of AQP4 reduction may be protective in acute severe hypoglycemia.

## Additional files

Additional file 1: ** Figure S1.** Identification of cultured rat astrocytes by immunofluorescence staining for GFAP and DAPI. Approximately 98% of the cells were GFAP-positive. *n* = 3 per group. (TIF 4592 kb)

Additional file 2: **Figure S2.** (A) The effects of GD on transendothelial permeability of monoculture and co-culture models in vitro. (B) The levels of claudin-5 protein in bEnd.3 cells were detected via Western blot. (C) Quantification of claudin-5 expression. *n* = 3 per group. (TIF 547 kb)

Additional file 3: **Figure S3.** The lactate content in the astrocyte culture medium. **p < 0.01 vs. the control + 5.5 mM glucose group. *n* = 3 per group. (TIF 268 kb)

## Abbreviations

AQP4: Aquaporin-4
AST: Astrocyte
BBB: Blood-brain barrier
BMECs: Brain microvascular endothelial cells
DAPI: 4′,6-Diamidino-2-phenylindole
EB: Evans blue
ELISA: Enzyme-linked immunosorbent assay
GD: Glucose deprivation
GFAP: Glial fibrillary acidic protein
NaF: Sodium fluorescein
PPAR-γ: Peroxisome proliferator-activated receptors γ
RT-PCR: Reversed transcription polymerase chain reaction
TJs: Tight junction proteins
