# Supplementary figures and images for: Aquaporin-4 deletion ameliorates hypoglycemia-induced BBB permeability by inhibiting inflammatory responses
Source: J Neuroinflammation. 2018 May 24;15:157. doi: 10.1186/s12974-018-1203-8 (PMC5968550; doi:10.1186/s12974-018-1203-8)

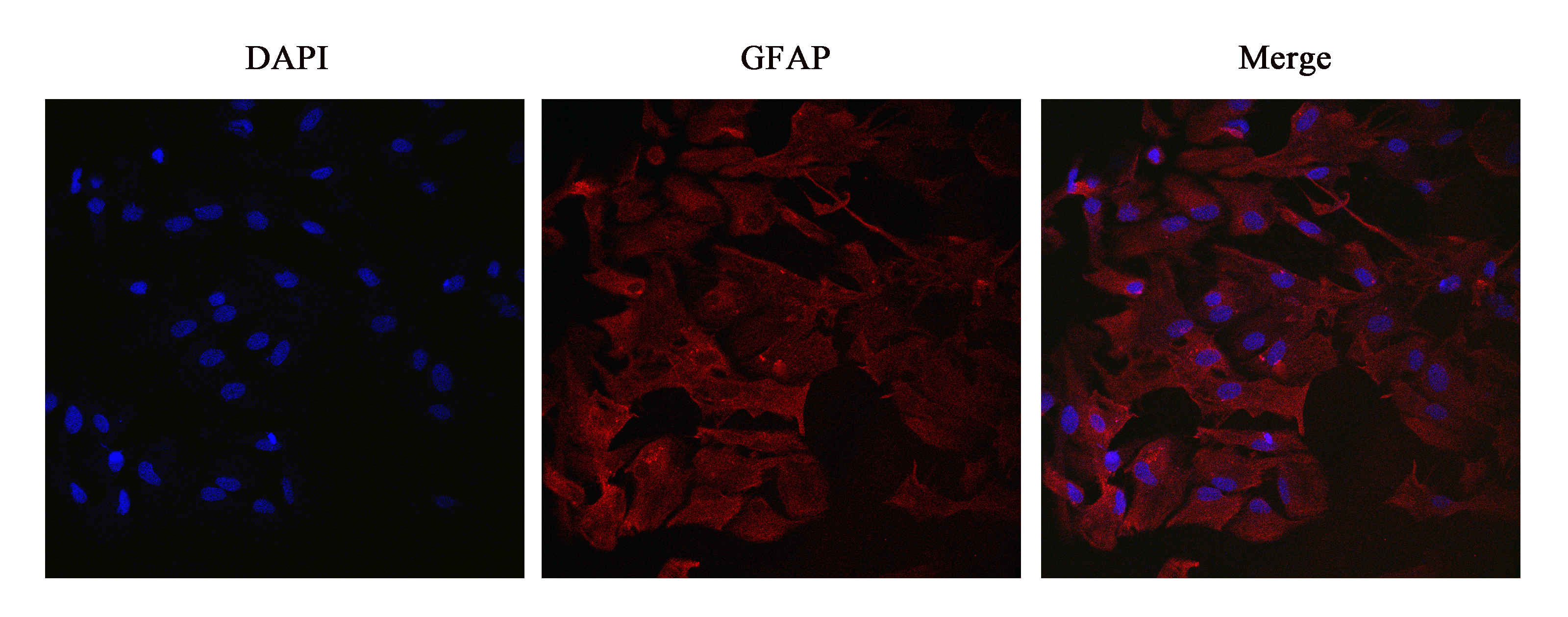

Supplement: Supplementary file 1 — Figure S1. Identification of cultured rat astrocytes by immunofluorescence staining for GFAP and DAPI. Approximately 98% of the cells were GFAP-positive. n = 3 per group. (TIF 4592 kb) [file 12974_2018_1203_MOESM1_ESM.tif]

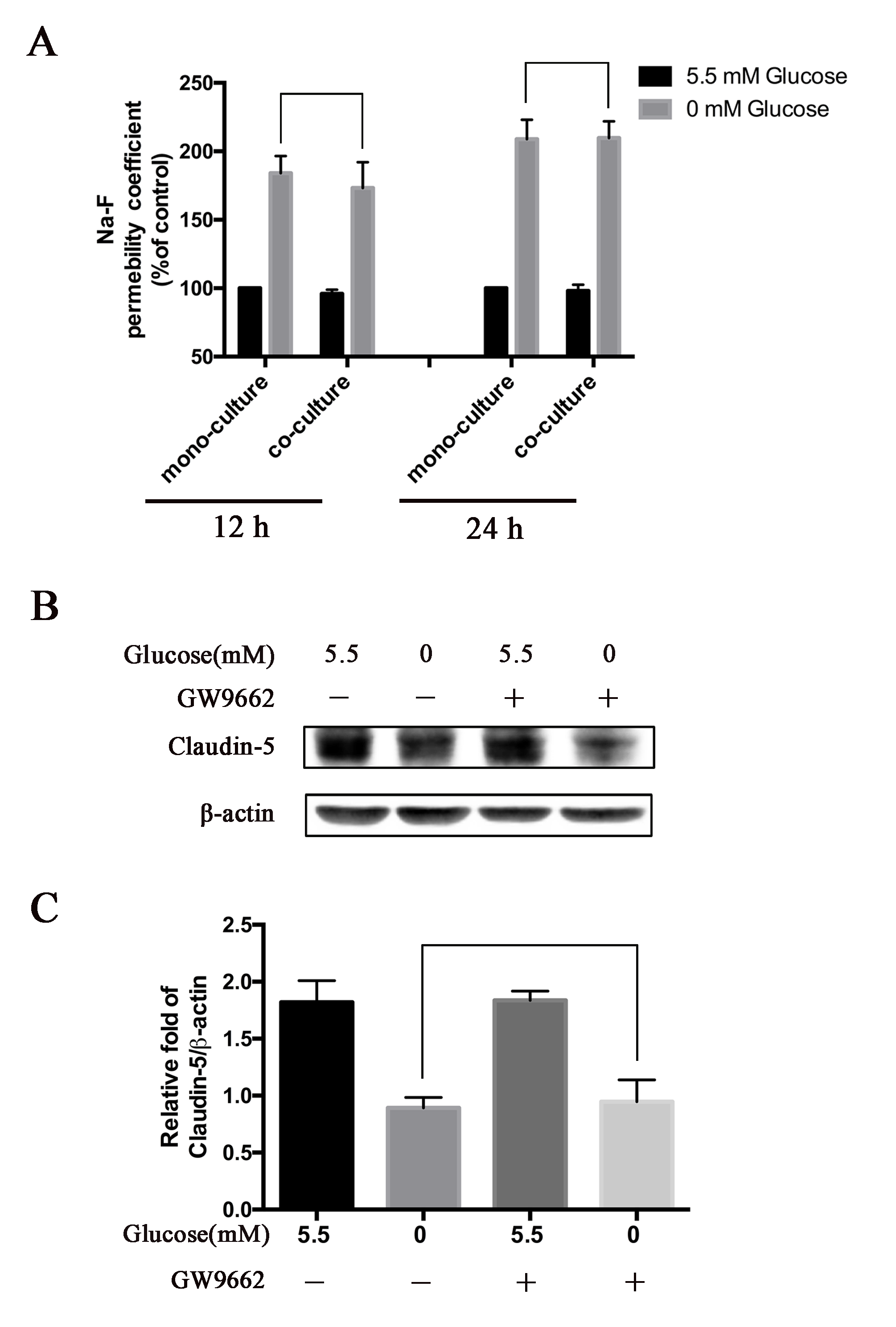

Supplement: Supplementary file 2 — Figure S2. (A) The effects of GD on transendothelial permeability of monoculture and co-culture models in vitro. (B) The levels of claudin-5 protein in bEnd.3 cells were detected via Western blot. (C) Quantification of claudin-5 expression. n = 3 per group. (TIF 547 kb) [file 12974_2018_1203_MOESM2_ESM.tif]

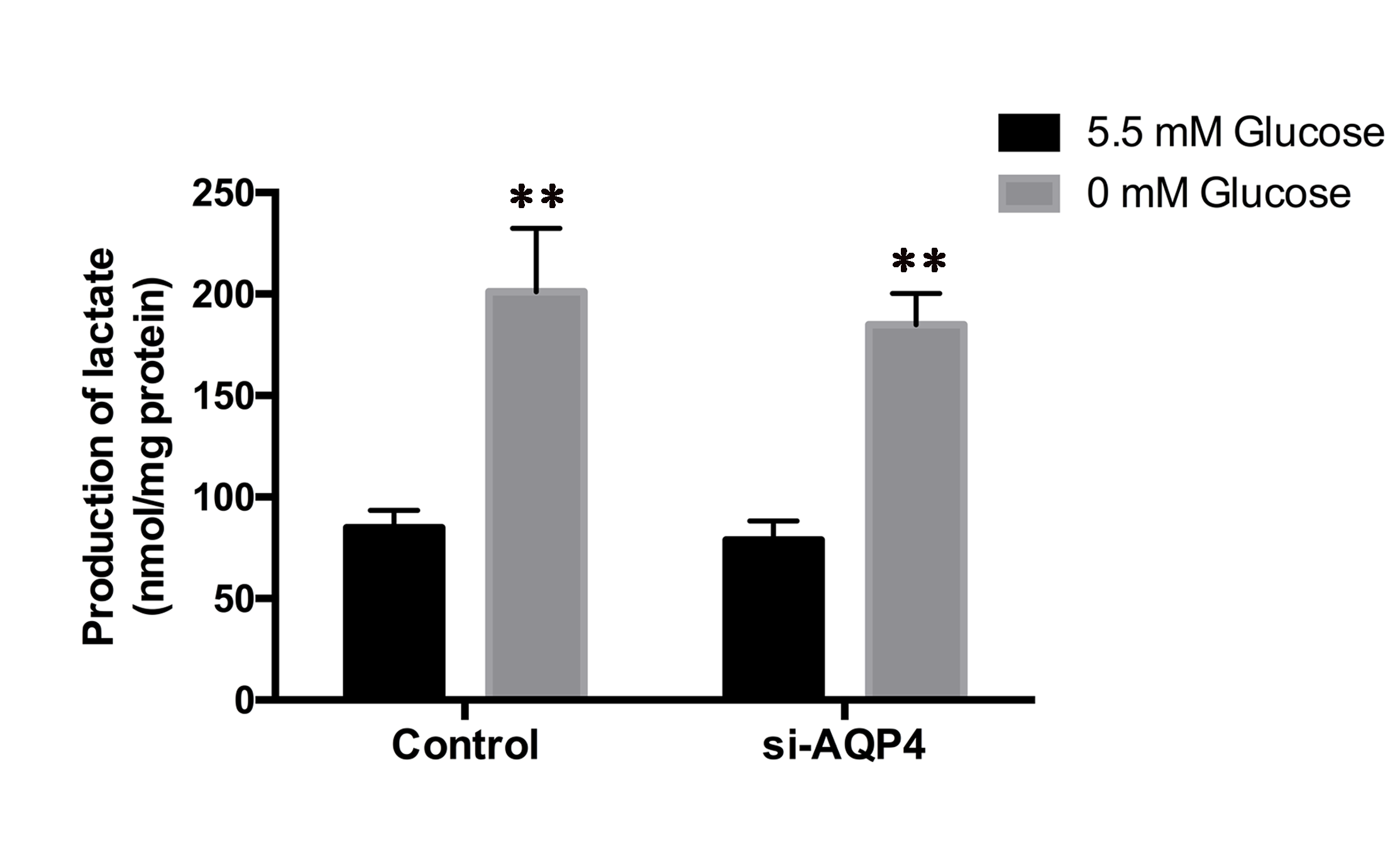

Supplement: Supplementary file 3 — Figure S3. The lactate content in the astrocyte culture medium. **p < 0.01 vs. the control + 5.5 mM glucose group. n = 3 per group. (TIF 268 kb) [file 12974_2018_1203_MOESM3_ESM.tif]
